# Longitudinal evaluation of corneal stability and tear film changes in first-time soft-contact lens wearers

**DOI:** 10.5935/0004-2749.2025-0118

**Published:** 2025-09-10

**Authors:** Neşe Arslan, Şule Barman Kakil, Bahar Aydoğdu, Hande Hüsniye Telek

**Affiliations:** 1 Department of Ophthalmology, Dışkapı Yıldırım Beyazıt Training and Research Hospital, Ankara, Turkey; 2 Department of Ophthalmology, Tayfur Ata Sökmen Faculty of Medicine, Hatay Mustafa Kemal University, Hatay, Turkey; 3 Department of Ophthalmology, Ankara Training and Research Hospital, Ankara, Turkey

**Keywords:** Contact lenses, hydrophilic, Cornea/surgery, Corneal diseases, Corneal topography, Adaptation, ocular/phy siology, Endo thelium, corneal/pathology, Refractive errors, Tears/me tabolism

## Abstract

**Purpose:**

Using advanced imaging techniques, this study aimed to evaluate corneal stability, epithelial remodeling, and tear film changes over a one-year period in first-time soft-contact lens wearers.

**Methods:**

A retrospective study was conducted on 100 eyes of 50 first-time daily soft-contact lens users aged 21–65 years with no prior rigid gas-permeable lens wear. The Sirius Scheimpflug imaging system was used to assess corneal topography, epithelial thickness, and non-invasive tear break-up time at baseline, 3, 6, and 12 months. Corneal warpage was evaluated using symmetry indices and Baiocchi Calossi Versaci indices. We performed statistical analysis using repeated-measures analyses of variance with Greenhouse-Geisser correction.

**Results:**

The mean baseline central corneal thickness was 537.83 (±7.92) µm, with no significant thinning after one year. The average simulated keratometry values remained stable, indicating no progressive corneal steepening or flattening. There were no significant changes in warpage indices over time, suggesting corneal shape preservation. Higher-order aberrations (coma, trefoil, and spherical aberrations) and non-invasive tear break-up time remained unchanged throughout the study period.

**Conclusions:**

Modern silicone hydrogel soft-contact lenses do not induce significant corneal warpage, epithelial remodeling, or optical aberrations over a one-year period. We found that corneal morphology and tear film stability were preserved, supporting the safety of soft-contact lens use. These findings provide clinically relevant insights into the long-term impact of contact lens wear. They may facilitate improved lens fitting strategies and preope rative refractive surgery assessments.

## INTRODUCTION

Soft-contact lenses (SCLs) are an appealing and con venient means of achieving clear vision. They are particularly popular among young adults. However, their long-term effects on corneal structure and biome chanics remain unclear. Understanding these effects is critical to ensuring safe lens wear and optimizing the outcomes of any future refractive surgery in wearers.

Maintaining corneal transparency is essential for vision and requires a stable oxygen supply to the cornea to support metabolic activity. However, contact lenses can reduce oxygen permeability, leading to a potentially hypoxic environment that may impact corneal metabolism^(1)^. Due to their high oxygen per meability, silicone hydrogel (SiH) has become the preferred lens material. These lenses minimize hypoxia-related complications and maintain corneal health^(2)^.

Corneal warpage is a condition that has been obser ved in individuals with prolonged soft-contact lens wear. It is characterized by central irregular astigmatism, loss of radial symmetry, and progressive steepening of the corneal curvature^(3)^. Such alterations have potential implications for refractive stability. Long-term lens wear is also a risk factor for corneal thinning and keratoconus progression^(4)^.

Recent advances in imaging technology allow for more precise evaluation of contact-lens-induced corneal changes. Together, Scheimpflug tomography, epi thelial thickness mapping, and tear film stability assessment can provide a detailed characterization of corneal morphology and biomechanics. This provides a deeper understanding of the effects of long-term contact lens use. Studies have shown that epithelial remodeling and tear film alterations may play a significant role in contact lens-related complications; yet, there is limited data on their combined impact over time^(5^-^7)^. Given that the epithelial thickness profile can serve as an early marker of corneal stress, its evaluation in contact lens users may provide valuable insight into subclinical structural changes.

We aimed to provide a longitudinal assessment of corneal thickness, astigmatism, warpage, and corneal aberrations in first-time soft-contact lens users. We used the Sirius Scheimpflug device to perform epithelial thickness mapping and tear film stability analysis, thereby providing a more comprehensive understanding of the corneal changes associated with lens wear. We also sought to evaluate whether any such changes are reversible and how they might affect the preoperative assessments of refractive surgery candidates. To the best of our knowledge, this is one of the first studies to analyze the combined effects of contact lens-induced warpage, epithelial remodeling, and ocular surface changes over an extended period.

## METHODS

This retrospective study was conducted on 100 eyes of 50 first-time daily SCL users (aged 21–65 years) with no prior rigid gas-permeable lens wear. None of the participants had previously used any type of contact lenses, including SCLs, rigid gas-permeable, or cosmetic lenses. Baseline ophthalmologic and topographic assessments were performed prior to the initial fitting and dispensing of lenses. We defined “first-time daily SCL users” as individuals who began the daily wear of SiH lenses (wearing time <14 hours/day) immediately after their baseline examination and adhered to regular use throughout the 12-month follow-up. Both eyes of each participant were included in the analysis to provide a comprehensive assessment of corneal stability and tear film changes. To account for the non-independence of data from two eyes belonging to the same individual, statistical analysis was performed in accordance with Armstrong’s guidelines for analyzing paired ocular data^(8)^.

The inclusion criteria were first-time daily soft-contact lens wear for a minimum of 6 hours per day over at least 3 months, aged between 21–65 years, a spherical prescription between −2.00 and −8.00 diopters (D), and a cylindrical value of ≤3.00 D. Only patients with no prior history of rigid gas-permeable (RGP) or hybrid lens use were included to exclude any pre-existing corneal remodeling from lens use. Baseline corneal assessments were conducted before initiating lens use to establish reference measurements.

The exclusion criteria were a history of ocular sur gery or trauma; corneal diseases such as keratoconus, dystrophies, autoimmune or collagen vascular diseases; allergies; the use of medications that affect corneal physiology, such as isotretinoin or amiodarone; a history of herpes keratitis; and diabetes mellitus. Patients who had previously worn multifocal or toric SCLs were also excluded to create a more homogenous study po pulation.

Each patient underwent a detailed ophthalmic evaluation, including visual acuity assessment, slit-lamp examination, and intraocular pressure measure ment with Goldmann applanation tonometry. The Sirius Scheimpflug device was used to determine corneal topo graphy and map epithelial thickness. Non-invasive tear break-up time (NITBUT) was assessed to identify any ocular surface changes. NITBUT was measured using the Sirius Scheimpflug Placido topographer (CSO, Florence, Italy) and its dedicated tear film analysis software module. Participants were instructed to blink twice and then refrain from blinking. The system then automatically recorded the time (in seconds) until the first distortion in the Placido rings occurred. The mean of three consecutive measurements was used in our analysis. Participants who reported using artificial tears were instructed to discontinue their use at least 24 hours prior to each imaging and measurement session. Compliance was confirmed verbally before each visit. Patients with active ocular surface diseases requiring continuous artificial tear use were excluded from the study. To ensure the accuracy of our corneal curvature measurements and to rule out any transient mechanical effects, we asked participants to discontinue contact lens wear for 8–12 hours before each examination.

Follow-up assessments were conducted 3-, 6-, and 12-months after the initiation of contact lens use. During these visits, visual acuity was reassessed, and anterior segment evaluations were repeated using the Sirius Scheimpflug imaging system. All measurements were taken at the same time of day (morning) to minimize the effects of diurnal variation on corneal thickness and curvature. Corneal warpage was evaluated using multiple parameters, including symmetry indices (SIf, SIb), Baiocchi Calossi Versaci indices (BCVf, BCVb), and epithelial thickness mapping.

### Contact lens characteristics and fitting assessment

Participants were fitted with commercially available daily-wear SiH contact lenses, including Acuvue Oasys^®^ (Johnson & Johnson Vision), Air Optix^®^ plus HydraGlyde (Alcon), and Biofinity^®^ (CooperVision). These lenses varied in their material properties, with oxygen per meability (Dk/t) values ranging from 86–175 ×10⁻⁹ (cm²/sec) (mLO₂/mL·mmHg), water content between 33%–48%, and modulus values between 0.72–1.10 MPa. Lens selection was based on lens availability and the participant’s refractive error and ocular surface status. Contact lens fit was evaluated using slit-lamp biomicroscopy, with which we assessed lens centration, coverage, movement on blink (ideally 0.2–0.4 mm), and push-up test responses. Lenses demonstrating excessive tightness or decentration were refitted using an alternative brand or base curve. Only participants with optimal lens fit at baseline were included in the study to minimize the potential for mechanical corneal deformation.

### Scheimpflug imaging: Sirius

Scheimpflug imaging was originally developed for military applications in 1904 by Captain Theodore Scheimpflug. It was later adapted for ophthalmology in the 1970s to assess cataract density. The Sirius system integrates a 360° rotating monochromatic Scheimpflug camera with a 22-ring Placido disk. It captures 25 radial sections of the cornea and anterior chamber. The system generates automatic measurements of anterior chamber volume and angle, providing detailed assessments of the corneal parameters. A single scan offers tangential and axial curvature readings of the front and back surfaces of the cornea, corneal refractive power, and pachymetric data. The combination of the Placido disk and the Scheimpflug images ensures accurate evaluations of anterior and posterior corneal structures. Previous studies have validated the accuracy and reliability of the corneal curvature and pachymetry measurements obtained using the Sirius device^(9^,^10)^.

### Statistical analysis

Data analysis was conducted using SPSS, v. 21.0 (IBM Corp., Armonk, NY, USA). Demographic data were summarized using counts and percentages, and means and standard deviations (SDs). Descriptive statistics included the mean, standard error of the mean (SEM), SDs, median, and range. Since both eyes of each participant were included in the analysis, a generalized estimating equation (GEE) approach was employed. This accounts for within-subject inter-eye correlations and prevents biased parameter estimation due to the non-independence of observations. GEE analysis confirmed that the observed changes in epithelial thickness, tear film stability (NITBUT), and higher-order aberrations across time points were not statistically significant, supporting the longitudinal stability of the corneal surface during soft-contact lens wear. To evaluate longitudinal changes, a repeated-measures analysis of variance was applied. Mauchly’s test was used to assess sphericity, and the Greenhouse-Geisser correction was applied when sphericity was violated. Additional statistical analysis was conducted using Stata 18 software, with a significance level set at p<0.05 for all comparisons.

## RESULTS

The study cohort included 50 first-time soft-contact lens users, consisting of 8 (16%) men and 42 (84%) women, with an average age of 29.37 (±SD) years (Table 1).

**Table 1:** Participant demographics

Variable	Value
Age (years), mean (±SD)	29.37 (±7.68)
Male, n (%)	8 (16%)
Female, n (%)	42 (84%)

SD= standard deviation.

### Corneal thickness and keratometry measurements

The mean baseline measurements of central corneal thickness (CCT) and apex corneal thickness were 537.83 (±SD) µm and 556.97 (±SD) µm, respectively. A slight, non-significant decrease in the mean CCT was observed after 1 year of SCL use (533.17 [±SD] µm, p=0.144).

Detailed values for these parameters at each time point are provided in table 2. The topographic simulated keratometry values, derived from Scheimpflug-based analysis, remained stable throughout the study period. SimK1 changed minimally from 43.04 (±SD) D at baseline to 43.00 (±SD) D at 12 months (p=0.544). SimK2 also showed a slight, non-significant increase from 44.19 (±SD) D to 44.31 (±SD) D (p=0.403). These findings suggest that SCLs do not induce progressive corneal steepening or flattening over time.

**Table 2:** Comparison of the corneal parameters of first-time soft-contact lens wearers at baseline and 3-, 6, and 12-month follow-up

Variable	Time	Mean (± SEM)	SD	Median (range)	p-value
Central corneal thickness (µm)	Baseline	537.83 (±7.92)	43.36	542 (416–614)	0.144
	3 months	533.83 (±6.97)	38.17	538 (426–10)	
	6 months	534.27 (±7.35)	40.26	545 (417–604)	
	1 year	533.17 (±7.4)	40.51	538.5 (418–610)	
Apex corneal thickness (µm)	Baseline	556.97 (±8.09)	44.31	561.5 (422–624)	0.914
	3 months	560.13 (±8.21)	44.98	568 (430–646)	
	6 months	559.53 (±10.44)	57.17	561 (420–719)	
	1 year	559.8 (±8.94)	48.95	558.5 (426–689)	
SimK1 (D)	Baseline	43.04 (±0.25)	1.36	42.96 (40.32–47.13)	0.544
	3 months	42.89 (±0.27)	1.46	42.82 (39.82–47.01)	
	6 months	42.99 ±0.25)	1.38	42.83 (40.02–47.19)	
	1 year	43 (± 0.24)	1.32	42.85 (41–47.15)	
SimK2 (D)	Baseline	44.19 (±0.26)	1.44	44.13 (41.1–49.04)	0.403
	3 months	44.13 (±0.3)	1.63	44.1 (40.6–48.85)	
	6 months	44.13 (±0.29)	1.57	43.87 (40.45–49.22)	
	1 year	44.31 (±0.24)	1.34	43.96 (42.01–49.02)	
Z22 (astigmatism)	Baseline	0.29 (±0.04)	0.23	0.24 (0.01–1.03)	0.456
	3 months	0.29 (±0.04)	0.2	0.26 (0.05–0.88)	
	6 months	0.26 (±0.03)	0.19	0.2 (0.03–0.68)	
	1 year	0.32 (±0.05)	0.27	0.23 (0.02–1.48)	
Z33 (trefoil)	Baseline	0.06 (±0.010	0.05	0.05 (0.01–0.2)	0.084
	3 months	0.11 (±0.02)	0.12	0.07 (0.01–0.49)	
	6 months	0.07 (±0.01)	0.04	0.07 (0.01–0.15)	
	1 year	0.08 (±0.01)	0.05	0.08 (0.02–0.28)	
Z31 (coma)	Baseline	0.05 (± 0.01)	0.04	0.03 (0.01–0.16)	0.134
	3 months	0.07 (±0.01)	0.08	0.05 (0–0.3)	
	6 months	0.05 (±0.01)	0.03	0.04 (0.02–0.15)	
	1 year	0.05 (±0.01)	0.04	0.03 (0.01–0.16)	
RMS	Baseline	0.12 (±0.01)	0.08	0.08 (0.03–0.3)	0.134
	3 months	0.16 (±0.03)	0.15	0.11 (0.05–0.63)	
	6 months	0.11 (±0.01)	0.07	0.1 (0.01–0.33)	
	1 year	0.12 (± 0.01)	0.07	0.1 (0.02–0.37)	
Spherical aberration	Baseline	0.05 (±0.02)	0.11	0.02 (0–0.57)	0.631
	3 months	0.04 (±0.01)	0.04	0.02 (0–0.18)	
	6 months	0.03 (±0.01)	0.04	0.02 (0.01–0.16)	
	1 year	0.05 (±0.02)	0.11	0.02 (0–0.6)	
SIf	Baseline	0.77 (±0.51)	2.54	0.01 (−0.85–9.39)	0.810
	3 months	0.71 (±0.52)	2.59	−0.10 (−1.29–9.83)	
	6 months	0.70 (±0.49)	2.44	0.06 (−1.48–9.27)	
	1 year	0.74 (±0.46)	2.28	0.15 (−1.06–8.83)	
SIb	Baseline	0.20 (±0.13)	0.67	0.03 (−0.23–2.39)	0.079
	3 months	0.16 (±0.13)	0.67	−0.02 (−0.36–2.45)	
	6 months	0.20 (±0.14)	0.70	0.01 (−0.19–2.63)	
	1 year	0.18 (±0.14)	0.68	0.02 (−0.29–2.45)	
BCVf	Baseline	0.52 (±0.26)	1.29	0.10 (0–5.18)	0.588
	3 months	0.51 (±0.26)	1.31	0.09 (0–5.41)	
	6 months	0.48 (±0.25)	1.23	0.10 (0–5.05)	
	1 year	0.50 (±0.23)	1.17	0.15 (0–4.92)	
BCVb	Baseline	0.52 (±0.29)	1.46	0.00 (0–5.74)	0.715
	3 months	0.51 (±0.29)	1.46	0.00 (0–5.85)	
	6 months	0.54 (±0.30)	1.52	0.00 (0–5.80)	
	1 year	0.54 (±0.30)	1.49	0.00 (0–5.81)	

BCVb= Baiocchi Calossi Versaci back; BCVf= Baiocchi Calossi Versaci front; D= diopters; RMS= root mean square; SD= standard deviation; SEM= standard error of the mean; Sib= symmetry index back; SIf= symmetry index front; SimK1= simulated flat keratometry; SimK2= simulated steep keratometry.

### Corneal warpage and surface irregularity

Corneal warpage was evaluated using symmetry indices (SIf, SIb), Baiocchi Calossi Versaci indices (BCVf, BCVb), and epithelial thickness mapping. The average SIf value decreased slightly from 0.77 (±SD) to 0.74 (±SD) over the course of a year, but the difference was not statistically significant (p=0.810). Similarly, SIb showed a minor decrease from 0.20 (±SD) to 0.18 (±SD), with no significant difference (p=0.079). The average BCVf value showed a marginal decline from 0.52 (±SD) to 0.50 (±SD), while the BCVb value increased slightly from 0.52 (±SD) to 0.54 (±SD). Again, neither of these changes was significant (p=0.588, p=0.715). Epithelial thickness mapping did not identify any regional thinning or irregularities that would indicate mechanical stress-induced shape alterations. The mean central epithelial thickness was 53.6 (±1.8) µm at baseline and 53.2 (±1.8) µm at the 12-month follow-up, with no statistically significant difference (p=0.096). Mea surements taken in the superior, inferior, nasal, and temporal regions also remained stable over the study period, with no significant changes observed. These findings are shown in table 3, which provides a detailed comparison of the average regional epithelial thickness values at baseline and 12 months. Overall, these findings suggest that properly fitted SCLs do not cause significant biomechanical alterations in the cornea over a year.

**Table 3:** Regional epithelial thickness in first-time soft-contact lens wearers at baseline and 12-month follow-up

Epithelial Region	Baseline (µm) mean (±SD)	12-Months (µm) mean (±SD)	p-value
Central	53.6 (±1.8)	53.2 (±1.8)	0.096
Superior	51.2 (±1.8)	51.0 (±1.8)	0.955
Inferior	55.1 (±1.8)	54.9 (±1.8)	0.366
Nasal	52.8 (±1.8)	52.5 (±1.8)	0.068
Temporal	54.3 (±1.8)	54.1 (±1.8)	0.793

SD= standard deviation.

### Higher-order aberrations and surface regularity

Our analysis of the higher-order aberrations trefoil, coma, and spherical aberrations found no significant changes across time points. The mean trefoil values were 0.06 (±SD) at baseline, 0.11 (±SD) at 3 months, 0.07 (±SD) at 6 months, and 0.08 (±SD) at 1 year (p=0.084). The coma values remained consistent at 0.05 (±SD) at both baseline and 1 year (p=0.134). The mean spherical aberration values showed no significant changes, measuring 0.05 (±SD) at baseline, 0.04 (±SD) at 3 months, 0.03 (±SD) at 6 months, and 0.05 (±SD) at 1 year (p=0.631). The root mean square (RMS) values also remained stable, suggesting that SCL wear does not significantly alter corneal wavefront properties. These results confirm that modern SiH lenses maintain optical quality without introducing clinically significant distortions.

### Tear film and ocular surface stability

NITBUT measurements remained stable throughout the follow-up period, suggesting that daily SCL wear does not induce substantial changes in ocular surface integrity or tear film stability. These findings further support the safety of modern SiH lenses and the main tenance of ocular surface homeostasis.

### Subgroup analysis by lens properties

A subgroup analysis was conducted to evaluate the effects of modulus, Dk/t, and water content on corneal parameters. However, no significant differences were observed between subgroups, indicating that the variations in lens material did not contribute to corneal changes within the first year of wear. This finding in dicates that modern SiH lenses are well-tolerated re gardless of material differences.

## DISCUSSION

SCLs are widely used for vision correction due to their superior optical performance and ease of use. However, their long-term effects on corneal morphology and biomechanics have not been fully elucidated. This study aimed to assess whether modern SiH lenses in duce corneal warpage, thickness alterations, or corneal aberrations.

Prolonged hypoxia and associated hypercapnia can lead to significant corneal alterations, including epithelial thinning, stromal edema, and endothelial dysfunction. While corneal changes are more commonly associated with RGP lenses, SCLs have also been implicated in minor refractive and topographic alterations, often due to corneal edema^(9^,^11^,^12)^. However, the introduction of SiH lenses, with their high Dk/t, has significantly reduced the reported incidences of hypoxia-related complications, including microcyst formation and limbal hyperemia. Previous studies suggest that contact lens wear may initially lead to corneal thickening, followed by gradual thinning over a prolonged period^(13^-^16)^. Liu and Pflugfelder found that individuals who had worn contact lenses for over 5 years had CCT reductions of 30–50 µm, with more pronounced thinning observed in those with RGP lenses. Possible explanations for this include increased tear osmolarity, elevated epithelial cell apoptosis, and chronic microtrauma induced by lens wear^(18)^. In contrast, we found that CCT remained stable over the first year of SCL use. We observed no statistically significant differences between pre-lens mea surements and those taken at 3, 6, and 12 months. This aligns with previous reports in which SiH lens wear did not induce permanent corneal thinning, and any minor alterations reverted after discontinuation^(18^,^19)^. Thus, modern SiH lenses appear to provide sufficient oxy genation to maintain corneal homeostasis over time.

In this study, we comprehensively evaluated the impact of SCL wear on corneal warpage and included epi thelial thickness mapping in our analysis. By integrating Scheimpflug tomography, epithelial thickness mapping, and tear film stability analysis, this study provides a more comprehensive assessment of the biomechanical effects of contact lens wear than has been performed in previous research. We provide a longitudinal evaluation of epithelial thickness changes and corneal stability over a year, offering a holistic perspective on contact lens-induced alterations. We found that no warpage occurred and corneal stability was maintained.

Although corneal warpage is usually associated with RGP lenses, it has also been reported in long-term soft-contact lens users. It is characterized by asymmetric corneal steepening and a loss of radial symmetry^(6^,^7^,^20)^. We evaluated symmetry indices (SIf, SIb) and Baiocchi Calossi Versaci indices (BCVf, BCVb), and performed epi thelial thickness mapping, but no statistically significant differences were found between baseline and follow-up values. This indicates that modern SCLs do not induce measurable corneal warpage in first-time users.

Tyagi et al. have reported that SiH lenses can cause anterior corneal flattening and posterior corneal steepening; however, their study analyzed the cornea immediately after lens removal^(21)^. In contrast, we incor porated an 8–12-hour lens-free interval prior to all assessments to ensure that our measurements were not influenced by transient mechanical effects. Our findings indicate that the shape of the cornea remains stable during long-term SiH lens wear, reinforcing their safety for continuous use.

Previous studies on SCL wear and higher-order aberrations have yielded conflicting results. Some have reported increased aberration rates due to myopia-correcting lenses^(22)^, whereas others found no significant differences^(23)^. We found that coma, trefoil, spherical aberrations, and RMS values remained stable across all time points, indicating that properly fitted SCLs do not induce significant optical distortions.

Tear film stability was also maintained throughout the study period, indicating that SCL wear does not compromise ocular surface homeostasis. This supports previous findings that modern SiH lenses, with their improved surface wettability, are better able to maintain tear film integrity compared to conventional hydrogel lenses^(24)^. Hence, SiH lenses are a viable option for individuals prone to dry eye symptoms or ocular surface instability.

Our findings demonstrate the safety of modern SiH lenses for daily wear. However, several aspects warrant further investigation. We followed participants for 1 year; future studies should examine long-term corneal changes over a longer period. Also, comparative evaluations of the effects of multifocal, toric, and RGP lenses would provide a more comprehensive understanding of contact lens-induced corneal changes. Future studies should also measure corneal hysteresis and corneal resistance to explore how lens wear in fluences corneal mechanical properties over time.

### Strengths and limitations

This study had several strengths, including its longi tudinal design and use of only first-time contact lens wearers, which prevented prior lens-induced corneal alterations from interfering with the results. The use of advanced imaging techniques such as Sirius topography, epithelial thickness mapping, and NITBUT analysis provided a comprehensive evaluation of corneal chan ges. Furthermore, the inclusion of a lens-free inter val before measurements excluded the influence of tran sient mechanical effects.

However, some limitations must be acknowledged. The relatively small sample size may have prevented the detection of subtle changes. Additionally, the follow-up duration was restricted to one year, so longer-term effects may not have been identified. Finally, the ex clusion of multifocal and toric lens users limited the generalizability of these findings to all soft lens wearers.

In conclusion, we found that modern SiH SCLs do not cause significant corneal warpage, thinning, or optical aberrations within the first year of use. Cor neal topographic stability was maintained across all eva luated parameters—including keratometry, sym metry indices, and regional epithelial thickness—demons trating the safety of these lenses for daily wear. Addi tionally, there was no increase in higher-order aberrations over the study period, and tear film integrity, assessed by NITBUT, was preserved, further supporting the ocular surface biocompatibility of SiH lenses. Subgroup analysis revealed no significant differences in outcomes attributable to the material properties of the lenses (Dk/t, modulus, or water content), indicating consistent safety profiles across different lens brands. Moreover, the inclusion of both eyes of each participant, analyzed using GEE methodology, enhanced the statistical robustness of our findings. Given that many contact lens users even tually pursue refractive surgery, understanding the long-term corneal effects of lens wear is essential. Our study underscores the importance of allowing a sufficient lens-free interval prior to topographic evaluation in sur gical candidates to avoid misinterpretation of results due to transient warpage. To the best of our knowledge, this study is one of only a few to longitudinally evaluate both keratometric changes and higher-order aberrations in first-time daily SCL wearers. Future research should incorporate larger populations, longer follow-ups, and a broader range of lens modalities to further elucidate the biomechanical effects of SCL use.

## Data Availability

The datasets used and analyzed during the current study are available from the corresponding author upon reasonable request.

## References

[1] McCanna DJ, Driot JY, Hartsook R, Ward KW (2008). Rabbit models of contact lens-associated corneal hypoxia: a
review of the literature. Eye Contact Lens..

[2] Dillahay SM (2007). Does the level of available oxygen impact comfort in contact lens
wear? A review of the literature. Eye Contact Lens..

[3] Wilson SE, Lin DT, Klyce SD, Reidy JJ, Insler MS (1990). Topographic changes in contact lens-induced corneal
warpage. Ophthalmology.

[4] Patrao LF, Canedo AL, Azevedo JL, Correa R, Ambrosio R (2016). Differentiation of mild keratoconus from corneal warpage
according to topographic inferior steepening based on corneal tomography
data. Arq Bras Oftalmol..

[5] Abtahi MA, Beheshtnejad AH, Latifi G, Akbari-Kamrani M, Ghafarian S, Masoomi A (2024). Corneal epithelial thickness mapping: a major
review. J Ophthalmol..

[6] Gurnani B, Kaur K (2025). Contact lens–related complications. 2023 Jun 11. StatPearls [Internet].

[7] Christy J, Gurnani B, Kaur K, Moutappa F (2020). Contact lens warpage: lost but found. Indian J Ophthalmol..

[8] Armstrong RA (2013). Statistical guidelines for the analysis of data obtained from one
or both eyes. Ophthalmic Physiol Opt..

[9] Henry VA, DeKinder JO, Bennett ES, Henry VA (2014). Soft lens material selection. Clinical manual of contact lenses.

[10] Chen W, McAlinden C, Pesudovs K, Wang Q, Lu F, Feng Y (2012). Scheimpflug-Placido topographer and optical low-coherence
reflectometry biometer: repeatability and agreement. J Cataract Refract Surg..

[11] Milla M, Pinero DP, Amparo F, Alio JL (2011). Pachymetric measurements with a new Scheimpflug photography-based
system: intraobserver repeatability and agreement with optical coherence
tomography pachymetry. J Cataract Refract Surg..

[12] Nichols JJ, Sinnott LT (2011). Tear film, contact lens and patient factors associated with
corneal staining. Invest Ophthalmol Vis Sci..

[13] Liesegang TJ (2002). Physiologic changes of the cornea with contact lens
wear. CLAO J..

[14] Kim YH, Lin MC, Radke CJ (2020). Limbal metabolic support reduces peripheral corneal edema with
contact-lens wear. Transl Vis Sci Technol..

[15] Lee JS, Park WS, Lee SH, Oum BS, Cho BM (2001). A comparative study of corneal endothelial changes induced by
different durations of soft contact lens wear. Graefes Arch Clin Exp Ophthalmol..

[16] Lei Y, Zheng X, Hou J, Xu B, Mu G (2015). Effects of long-term soft contact lens wear on corneal thickness
and corneal epithelial thickness of myopic subjects. Mol Med Rep..

[17] Liu Z, Pflugfelder SC (2000). The effects of long-term contact lens wear on corneal thickness,
curvature, and surface regularity. Ophthalmology.

[18] Gonzalez-Meijome JM, Gonzalez-Perez J, Cervino A, Yebra-Pimentel E, Parafita MA (2003). Changes in corneal structure with continuous wear of high-Dk soft
contact lenses: a pilot study. Optom Vis Sci..

[19] Yebra-Pimentel E, Giraldez MJ, Arias FL, Gonzalez J, Gonzalez JM, Parafita MA (2001). Rigid gas permeable contact lens and corneal
topography. Ophthal Physiol Opt..

[20] Stapleton F, Bakkar M, Carnt N, Chalmers R, Vijay AK, Marasini S (2021). CLEAR - contact lens complications. Cont Lens Anterior Eye..

[21] Tyagi G, Collins M, Read S, Davis B (2010). Regional changes in corneal thickness and shape with soft contact
lenses. Optom Vis Sci..

[22] Roberts B, Athappilly G, Tinio B, Naikoo H, Asbell P (2006). Higher order aberrations induced by soft contact lenses in normal
eyes with myopia. Eye Contact Lens..

[23] Berntsen DA, Merchea MM, Richdale K, Mack CJ, Barr JT (2009). Higher-order aberrations when wearing sphere and toric soft
contact lenses. Optom Vis Sci..

[24] Schafer J, Steffen R, Reindel W, Chinn J (2015). Evaluation of surface water characteristics of novel daily
disposable contact lens materials, using refractive index shifts after
wear. Clin Ophthalmol..

